# Panel-based next-generation sequencing with copy number variant detection pipeline could increase the detection rate of hereditary colorectal cancer syndrome compared with Sanger sequencing

**DOI:** 10.1186/s13053-026-00350-1

**Published:** 2026-08-07

**Authors:** Joonsang Yu, Jaeyeon Ryu, Sollip Kim, Jihun Kim, Yong Sang Hong, Woochang Lee, Seok-Byung Lim, Jeong-Sik Byeon, Sail Chun

**Affiliations:** 1 Department of Laboratory Medicine, University of Ulsan College of Medicine and Asan Medical Center, Seoul, Korea; 2 Department of Pathology, University of Ulsan College of Medicine and Asan Medical Center, Seoul, Korea; 3 Department of Oncology, University of Ulsan College of Medicine and Asan Medical Center, Seoul, Korea; 4 Division of Colon and Rectal Surgery, Department of Surgery, University of Ulsan College of Medicine and Asan Medical Center, Seoul, Korea; 5 Department of Gastroenterology, University of Ulsan College of Medicine, Asan Medical Center, Seoul, Korea

**Keywords:** Colorectal cancer, Hereditary colorectal cancer, Lynch syndrome, Next-generation sequencing, Sanger sequencing, Mismatch repair genes

## Abstract

**Background:**

Hereditary colorectal cancer syndromes, such as Lynch syndrome and familial adenomatous polyposis, arise from pathogenic/likely pathogenic (P/LP) germline variants in DNA mismatch repair or tumor suppressor genes. Traditional Sanger sequencing could cover only phenotype-driven genes and cannot detect copy number variants (CNVs). Next-generation sequencing (NGS) enables simultaneous multi-gene analysis and CNV detection. In this study, we compared the results of phenotype-driven Sanger sequencing with those of panel-based NGS in patients suspected of having hereditary colorectal cancer syndromes at a single institution.

**Methods:**

Patients tested for hereditary colorectal cancer syndromes between 2008 and 2018 (Sanger) and 2019–2022 (NGS) were retrospectively analyzed. The NGS assay targeted 171 cancer predisposition genes, and CNVs were inferred by read-depth analysis and confirmed with multiplex ligation-dependent probe amplification. Detected variants were classified per ACMG/AMP 2015 guidelines. Microsatellite instability (MSI) and mismatch repair (MMR) immunohistochemistry (IHC) results were compared with molecular findings.

**Results:**

Among 423 patients (254 Sanger, 169 NGS), the detection rate of pathogenic or likely pathogenic variants was higher in NGS (55.0%) than Sanger sequencing (40.6%). NGS additionally identified 17 CNVs (18.1%) that were undetectable by Sanger sequencing and revealed incidental P/LP variants in other cancer-related genes. MSI and MMR IHC results showed strong concordance with molecular findings.

**Conclusion:**

Panel-based NGS with CNV analysis was associated with a higher detection rate of clinically relevant variants than phenotype-driven Sanger sequencing in this single-institution cohort. These findings support the clinical utility of comprehensive germline testing for patients suspected of having hereditary colorectal cancer syndromes, particularly when CNV detection or simultaneous multi-gene analysis is required.

**Supplementary Information:**

The online version contains supplementary material available at https://doi.org/10.1186/s13053-026-00350-1.

## Introduction

Colorectal cancer is the third most commonly diagnosed cancer and the second leading cause of cancer-related death worldwide [1]. Its risk factors are older age, male gender, family history of colorectal cancer, known hereditary cancer-predisposing syndromes, obesity, diabetes, high consumption of red and processed meat, low fiber intake, high alcohol intake, and smoking [2, 3]. Although most colorectal cancer cases are sporadic and caused by lifestyle factors, approximately 10%–33% of colorectal cancer cases in individuals aged below 50 years are hereditary [3, 4]. Hereditary colorectal cancer syndrome includes Lynch syndrome, familial adenomatous polyposis (FAP), Peutz–Jeghers syndrome, MUTYH-associated syndrome, Cowden syndrome, and juvenile polyposis syndrome [2–8]. They are associated with pathogenic or likely pathogenic (P/LP) variants in genes such as *MLH1*, *MSH2*, *MSH6*, *EPCAM*, and *PMS2* (Lynch syndrome); *APC* (FAP); *STK11* (Peutz–Jeghers syndrome); *MUTYH* (MUTYH-associated syndrome); *PTEN* (PTEN hamartoma tumor syndrome and Cowden syndrome); and *BMPR1A* and *SMAD4* (Juvenile polyposis syndrome). As such, hereditary colorectal cancer syndrome should be diagnosed by identifying important variants in these genes; furthermore, colorectal cancer should be prevented and treated early through education, cancer screenings, and familial genetic tests [2–8]. Traditionally, Sanger sequencing has been used for genetic testing; when hereditary colorectal cancer syndrome is suspected, only specific genes are tested according to phenotype. Since the introduction of next-generation sequencing (NGS), panel-based testing has enabled the simultaneous testing of related genes; thus, comprehensive genetic testing can be performed at once. Copy number variants (CNVs) may also be detected by read-depth analysis of NGS [9–12]. In this study, we compared the results obtained from previously used Sanger sequencing with those determined through NGS, which is currently being used, at a single institution.

## Materials and methods

### Study patients

Patients diagnosed with colorectal cancer or colon polyps and suspected to have hereditary colorectal cancer syndromes were enrolled for genetic analysis. Two study periods were defined: January 2008 to December 2018 for Sanger sequencing and April 2019 to December 2022 for NGS. The patients were categorized into several groups based on clinical suspicion and family history: FAP, Lynch syndrome, Peutz–Jeghers syndrome, Cowden syndrome, and others. This study was approved by the Institutional Review Board of Asan Medical Center, Seoul, Korea (approval No.: 2025 − 1252).

### Sanger sequencing

Genomic DNA was extracted from peripheral blood leukocytes in accordance with standardized protocols. *APC*, *MLH1*, *MSH2*, *STK11*, and *PTEN* were amplified using in-house primers targeting all coding exons and adjacent intronic regions. Sequencing reactions were performed using the BigDye Terminator v3.1 cycle sequencing kit (Applied Biosystems, Foster City, CA, USA) on an ABI 3730xl DNA analyzer (ThermoFisher scientific, Waltham, Massachusetts, USA).

### NGS

A hereditary tumor panel comprising 171 genes associated with cancer predisposition was used for patients undergoing NGS. Libraries were prepared using a Dxome target enrichment kit (Seongnam, Korea) and sequenced on a MiSeqDx platform (Illumina, San Diego, CA, USA). The nucleotide sequencing results obtained from MiSeqDx were processed using the built-in software MiSeq Reporter (Illumina Inc.) for demultiplexing. They were subsequently converted into FASTQ files that were analyzed using the DxSeq NGS Gene Analysis System (Dxome). The CNVs suspected during NGS analysis and indicated by aberrant read depths were validated using multiplex ligation-dependent probe amplification (MLPA) assays (MRC Holland, Amsterdam, the Netherlands) [11, 12].

### Clinical interpretation of sequence variants

All identified variants were classified as pathogenic, likely pathogenic, variants of uncertain significance, or benign/likely benign in accordance with the 2015 American College of Medical Genetics and Genomics and the Association for Molecular Pathology (AMP) guidelines [13].

### Microsatellite instability (MSI) test and immunohistochemical (IHC) staining

The results of MSI testing and IHC staining for mismatch repair (MMR) proteins (MLH1, MSH2, MSH6, and PMS2) in the tissue samples of all patients were collected and compared with the sequencing results. The MMR status was determined through either PCR fragment analysis or IHC. For PCR fragment analysis, formalin-fixed, paraffin-embedded (FFPE) tissue sections were prepared and tumor area was marked to achieve more than 40% tumor cell fraction. Three to six unstained tissue sections were scraped along previously marked tumor and non-tumor area and then DNA was extracted. Primers were used to amplify five standard microsatellite markers: BAT-25 and BAT-26 for mononucleotide repeats, and D5S346, D2S123, and D17S450 for dinucleotide repeats [14–16]. The findings of MSI testing were interpreted using the following classifications: MSI-high when MSI was detected in ≥ 2 loci; MSI-low when MSI was found in only one locus; and MSI-stable when no instability was observed.

For IHC staining, FFPE tissue sections were processed using a BenchMark XT automatic immunostaining device (Ventana Medical Systems, Tucson, AZ, USA) with OptiView DAB IHC Detection Kit (Ventana Medical Systems, Tucson, AZ, USA) according to the manufacturer’s instructions. Four µm-thick sections obtained with a microtome, which were transferred onto silanized charged slides and allowed to dry for 10 min at room temperature, followed by 20 min in an incubator at 65 °C. Sections were performed by heat-induced epitope retrieval (HIER) method using Cell Conditioning 1 (CC1) buffer for 32 min (MSH2 and MSH6) or 64 min (MLH1 and PMS2) and incubated for 16 min (MSH2, MSH6 and PMS2) or 32 min (MLH1) with antibodies in the autoimmunostainer. Antigen-antibody reactions were visualized using Ventana OptiView DAB IHC Detection Kit (Optiview HQ Linker 8 min, Optiview HRP Multimer 8 min, Optiview H2O2/DAB 8 min, and Optiview Copper 4 min). Counterstaining was performed by using Ventana Hematoxylin II for 12 min and Ventana Bluing reagent for 4 min. Finally, all slides are removed from the stainer, dehydrated, and cover slipped for microscopic examination. Complete loss of nuclear staining with clearly discernible internal positive controls was scored as loss of expression. For interpretation, MLH1 and PMS2 were evaluated as a pair, and MSH2 and MSH6 were evaluated as another pair, reflecting the functional heterodimeric relationships of these proteins [17–20].

## Results

A total of 254 and 169 patients were enrolled from Sanger sequencing and NGS, respectively (Table 1). The Sanger sequencing group included 135 males and 119 females, with a median age of 43.6 years; the NGS group included 89 males and 80 females, with a median age of 44.6 years. In Sanger sequencing, the patients were classified as follows: 111 in the FAP group, 135 in the Lynch syndrome group, 3 in the Peutz–Jeghers syndrome group, and 5 in the Cowden syndrome group. In NGS, the patients were categorized as follows: 62 in the FAP group, 83 in the Lynch syndrome group, 3 in the Peutz–Jeghers syndrome group, 11 in the Cowden syndrome group, and 10 in others group. The total number of Sanger sequencing testing was 254, with an average of 23.1 per year; the total number of NGS testing was 169, with an average of 42.3 per year.

**Table 1:** Demographic table

	Sanger sequencing	Next-generation sequencing
**Total**	254	169
**Gender**		
Male	135	89
Female	119	80
**Age**		
0–9	2	2
10–19	10	8
20–29	46	17
30–39	52	37
40–49	48	37
50–59	50	37
60–69	30	23
70+	16	8
**Group**		
FAP	111	62
Lynch syndrome	135	83
Peutz-Jeghers syndrome	3	3
Cowden syndrome	5	11
Others	-	10

Abbreviations: FAP, familial adenomatous polyposis

**Fig. 1:**
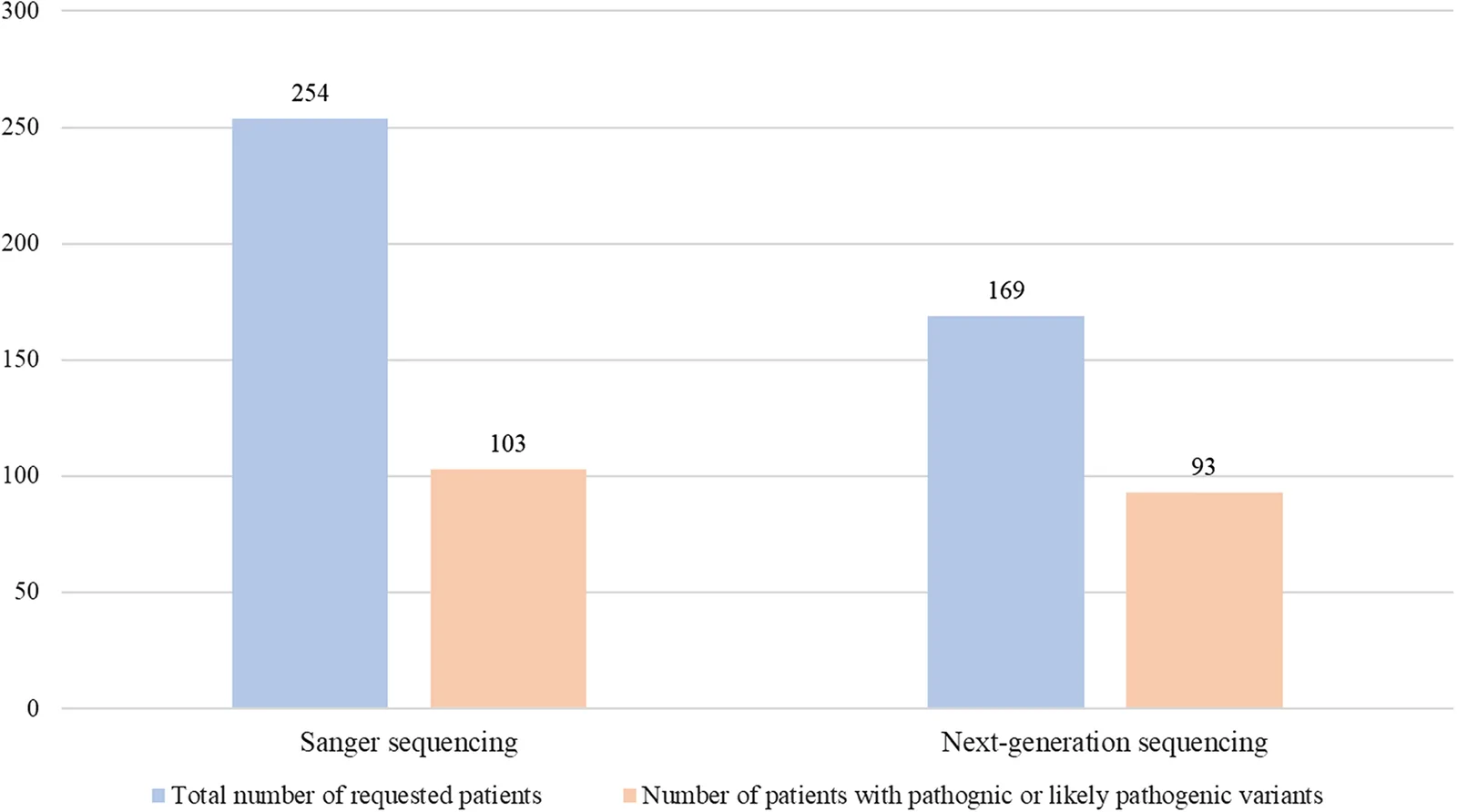
Total number of patients who were requested a genetic test for hereditary colorectal cancer and the number of patients who had the pathogenic or likely pathogenic variant detected

The detection rates of P/LP variants were 40.6% and 55.0% for Sanger sequencing and NGS, respectively (Fig. 1). When stratified by clinical disease category, the detection rates of pathogenic or likely pathogenic variants by Sanger sequencing were 23.0% in the Lynch syndrome group, 61.3% in the FAP group, 66.7% in the Peutz–Jeghers syndrome group, and 40.0% in the Cowden syndrome group. For NGS, the corresponding detection rates were 57.8% in the Lynch syndrome group, 54.8% in the FAP group, 66.7% in the Peutz–Jeghers syndrome group, 72.7% in the Cowden syndrome group, and 10.0% among other patients referred for hereditary colorectal cancer syndrome testing.

**Fig. 2:**
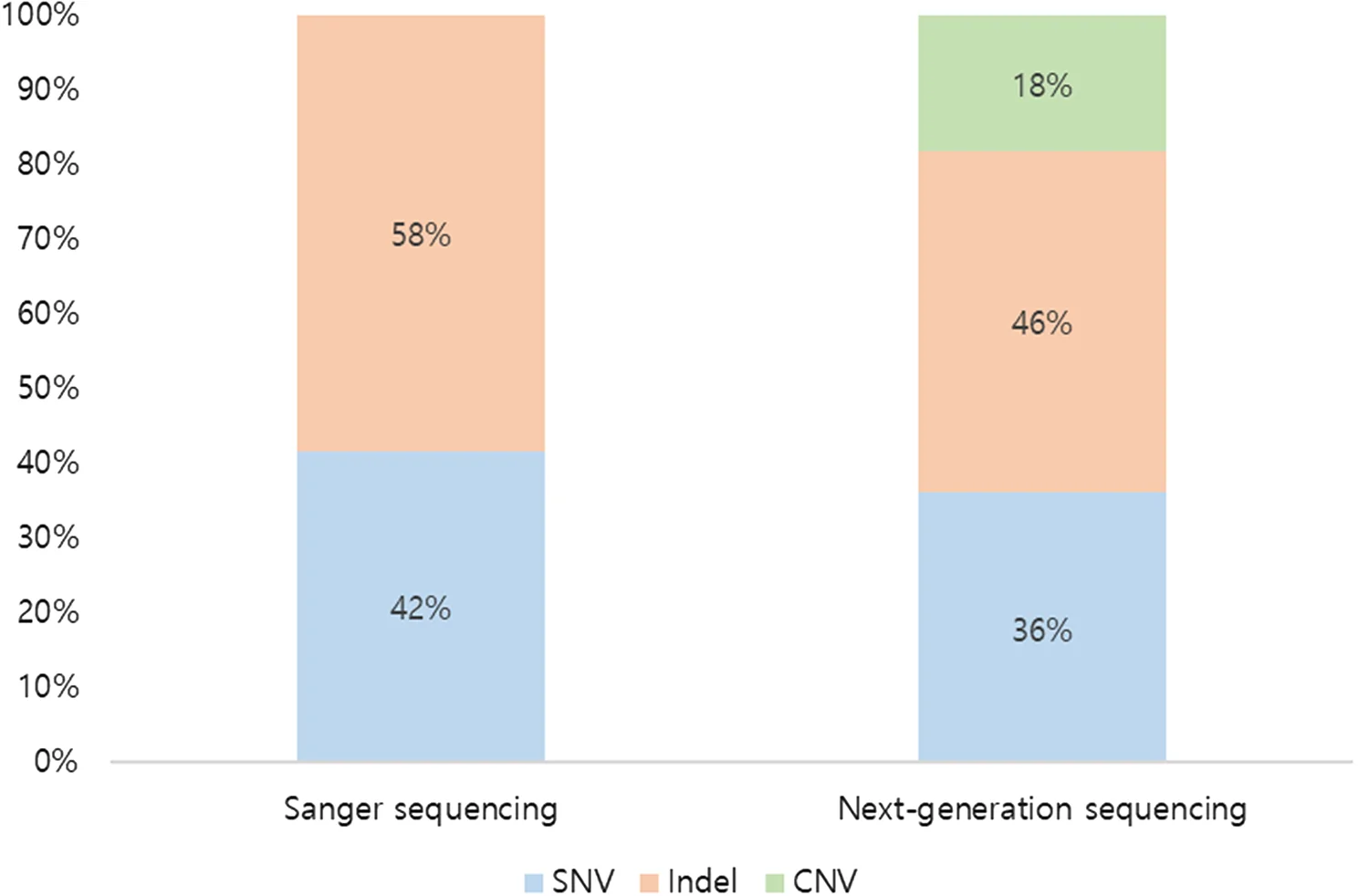
Total number of pathogenic or likely pathogenic variants and the fraction of variant types in Sanger sequencing and next-generation sequencing. Abbreviations: SNV, single nucleotide variant; CNV, copy number variant

In Sanger sequencing, 43 SNV variants (41.7%) and 60 indels (58.3%) were detected (Fig. 2). In NGS, 34 SNV variants (36.2%), 43 indel variants (45.7%), and 17 CNVs (18.1%) were determined (Table 2). In NGS, apart from the suspected diseases, variants in other genes were found in several patients. Specifically, in patients suspected of having Lynch syndrome, two variants were identified in *APC* and *BRCA2*. One patient with an *APC* variant also had a *BRCA1* variant. Additionally, one variant was detected in *MUTYH*. Among patients diagnosed with colorectal cancer but without a specific hereditary syndrome, one case had a *BRCA1* variant (Table 3). Among patients with incidental *BRCA*1/2 P/LP variants, review of available family history information showed a family history of breast and ovarian cancer in one patient, colorectal cancer in one patient, gastric cancer in one patient, and no documented family history of cancer in one patient.

**Table 2:** Copy number variants (CNVs) identified in this study and their nomenclature Abbreviations: HGVS, Human Genome Variation Society

Gene	HGVS nomenclature
*APC*	NM_000038.5 (*APC*):c.(?_-30253)_ (*26_? )del
*APC*	NM_000038.5 (*APC*):c.(531+1_532-1)_ (645+1_646-1)del
*APC*	NM_000038.5 (*APC*):c.(422+1_423-1)_ (531+1_532-1)del
*APC*	NM_000038.5 (*APC*):c.(-29995_-27)_(*26_? )del
*APC*	NM_000038.5 (*APC*):c.(933+1_934-1)_(1312+1_1313-1)del
*MLH1*	NM_000249.3 (*MLH1*):c.(?_-198)_(116+1_117-1)del
*MLH1*	NM_000249.3 (*MLH1*):c.(?_-198)_(116+1_117-1)del
*MLH1*	NM_000249.3 (*MLH1*):c.(545+1_546-1)_(790+1_791-1)del
*MSH2*	NM_000251.3 (*MSH2*):c.(?_-36)_(1076+1_1077-1)del
*MSH2*	NM_000251.3 (*MSH2*):c.(2005+1_2006-1)_(2210+1_2211-1)del
*MSH2*	NM_000251.3 (*MSH2*):c.(1661+1_1662-1)_(1759+1_1760-1)del
*MSH2*	NM_000251.3 (*MSH2*):c.(?_-36)_(1076_? )del
*MSH2*	NM_000251.3 (*MSH2*):c.(?_-36)_(211+1_212-1)del
*MSH2*	NM_000251.3 (*MSH2*):c.(1276+1_1277-1)_(1661+1_1662-1)del
*MSH2*,* EPCAM*	NC_000002.11:g.(?_47596618)_(47643594_? )del
*PTEN*	NM_000314.4 (*PTEN*):c.(209+1_210-1)_(*26_? )del
*STK11*	NM_00455.5 (*STK11*):c.(?_-28)_c.(*16+10G_? )del

Abbreviations: HGVS, Human Genome Variation Society

**Table 3:** Incidental findings identified by next-generation sequencing

Genes	Numbers	Variants list
*APC	2	NM_000038.5 (*APC*):c.783_786delTGAA (p.Asn261Lysfs*31) NM_000038.5 (*APC*):c.1072 C > T (p.Gln358*)
*BRCA1*	2	NM_007294.3 (*BRCA1*):c.5445G > A(p.Trp1815*) NM_007294.3 (*BRCA1*):c.5496_5506delinsA (p.Val1833Serfs*7)
*BRCA2*	2	NM_000059.3 (*BRCA2*):c.9076 C > T (p.Gln3026*) NM_000059.3 (*BRCA2*):c.1399 A > T (p.Lys467*)
†*MSH6*	3	NM_000179.2 (*MSH6*):c.885dupA (p.Val296Serfs*16) NM_000179.2 (*MSH6*):c.3261dupC (p.Phe1088Leufs*5) NM_000179.2 (*MSH6*):c.2150_2153delTCAG (p.Val717Alafs*18)
†*PMS2*	3	NM_000535.5 (*PMS2*):c.1738 A > T (p.Lys580*) NM_000535.5 (*PMS2*):c.164-1G > C (p.?) NM_000535.5 (*PMS2*):c.164-1G > C (p.?)
‡*PTEN*	1	NM_000314.4 (*PTEN*):c.773delT (p.Phe258Serfs*8)

* Variants of the *APC* gene were found in patients of Lynch group

† Variants of *MSH6* and *PMS2* genes were found in patients of Lynch group, but the Sanger sequencing for these genes was unavailable in our institution

‡ A variant of *PTEN* gene was found in a patient of familial adenomatous polyposis group

Among the patients suspected of having Lynch syndrome, those who underwent MSI testing included 107 and 63 patients in the Sanger sequencing and NGS groups, respectively. Furthermore, 115 and 39 patients underwent IHC staining for MMR proteins, respectively.

In the Sanger sequencing group, the detection rates for P/LP variants of MMR genes were 59.1% (26/44), 0% (0/5), and 1.7% (1/58) in the MSI-high, MSI-low, and MSI-stable groups, respectively. In the next-generation sequencing (NGS) group, the corresponding detection rates were 61.7% (29/47) in the MSI-high group, 100% (2/2) in the MSI-low group, and 7.1% (1/14) in the MSI-stable group.

When stratified according to MMR protein IHC staining results, P/LP variants were identified in 65.0% (13/20) of cases with MLH1/PMS2 loss, 60.9% (14/23) with MSH2/MSH6 loss, 33.3% (1/3) with concurrent loss of both MMR protein pairs (MLH1/PMS2 and MSH2/MSH6), and 2.9% (2/69) with intact MMR expression in the Sanger sequencing group. In the NGS group, P/LP variants were identified in 68.8% (11/16) of cases with MLH1/PMS2 loss, 86.7% (13/15) with MSH2/MSH6 loss, and 25.0% (2/8) with intact MMR expression. Among cases with MLH1/PMS2 loss, P/LP variants involving *MLH1* or *PMS2* were identified in 65.0% (13/20) of cases in the Sanger sequencing group and 62.5% (10/16) of cases in the NGS group. Among cases with MSH2/MSH6 loss, P/LP variants involving *MSH2*, *MSH6*, or *EPCAM* were identified in 60.9% (14/23) of cases in the Sanger sequencing group and 86.7% (13/15) of cases in the NGS group. In cases with intact MMR expression, the detected P/LP variants involved BRCA2 rather than genes encoding MMR proteins.

## Discussion

This retrospective study compared the results obtained by the switching testing method from Sanger sequencing with those from NGS for hereditary colorectal cancer syndrome at a single institution. The results showed that the detection rate of P/LP variants increased from 40.6% with Sanger sequencing to 55.0% with NGS. In the four groups classified according to tissue biopsy and medical records, namely, Lynch syndrome group, Peutz–Jeghers syndrome group, Cowden syndrome group, and others, the detection rate of NGS was higher than that of Sanger sequencing. However, in the FAP group, the detection rate of Sanger sequencing was higher than that of NGS. This difference was probably due to the increased use of genetic testing and the higher number of referrals during the implementation period of NGS than that of Sanger sequencing.

Sanger sequencing has been used as the gold standard for detecting germline variants that cause colorectal cancer. Although this technique examines only the specific genes related to suspected diseases, NGS involves the use of a multi-gene panel, thereby enabling the simultaneous testing of multiple genes. Because hereditary colorectal cancer syndromes, particularly polyposis conditions, exhibit a considerable phenotypic overlap, testing with a multi-gene panel is recommended [5, 20, 21]. In the present study, 171 genes related to hereditary tumors were tested at once; in 13 cases, P/LP variants were found in genes other than the suspected ones (Table 3), including *BRCA1*/*2* P/LP variants. The variable family history observed among patients with incidental BRCA1/2 P/LP variants further underscores the importance of family history assessment and appropriate genetic counseling when incidental P/LP variants are identified by multi-gene panel testing. Consistent with previous reports, our findings support the genetic and phenotypic heterogeneity of hereditary colorectal cancer syndromes, in which clinical phenotypes do not always correspond precisely to specific causative genes [22, 23]. In this context, our results further support the clinical utility of multi-gene panel NGS as a complementary approach to phenotype-driven Sanger sequencing, particularly by enabling simultaneous analysis of multiple genes in patients with suspected hereditary colorectal cancer syndromes.

**Fig. 3:**
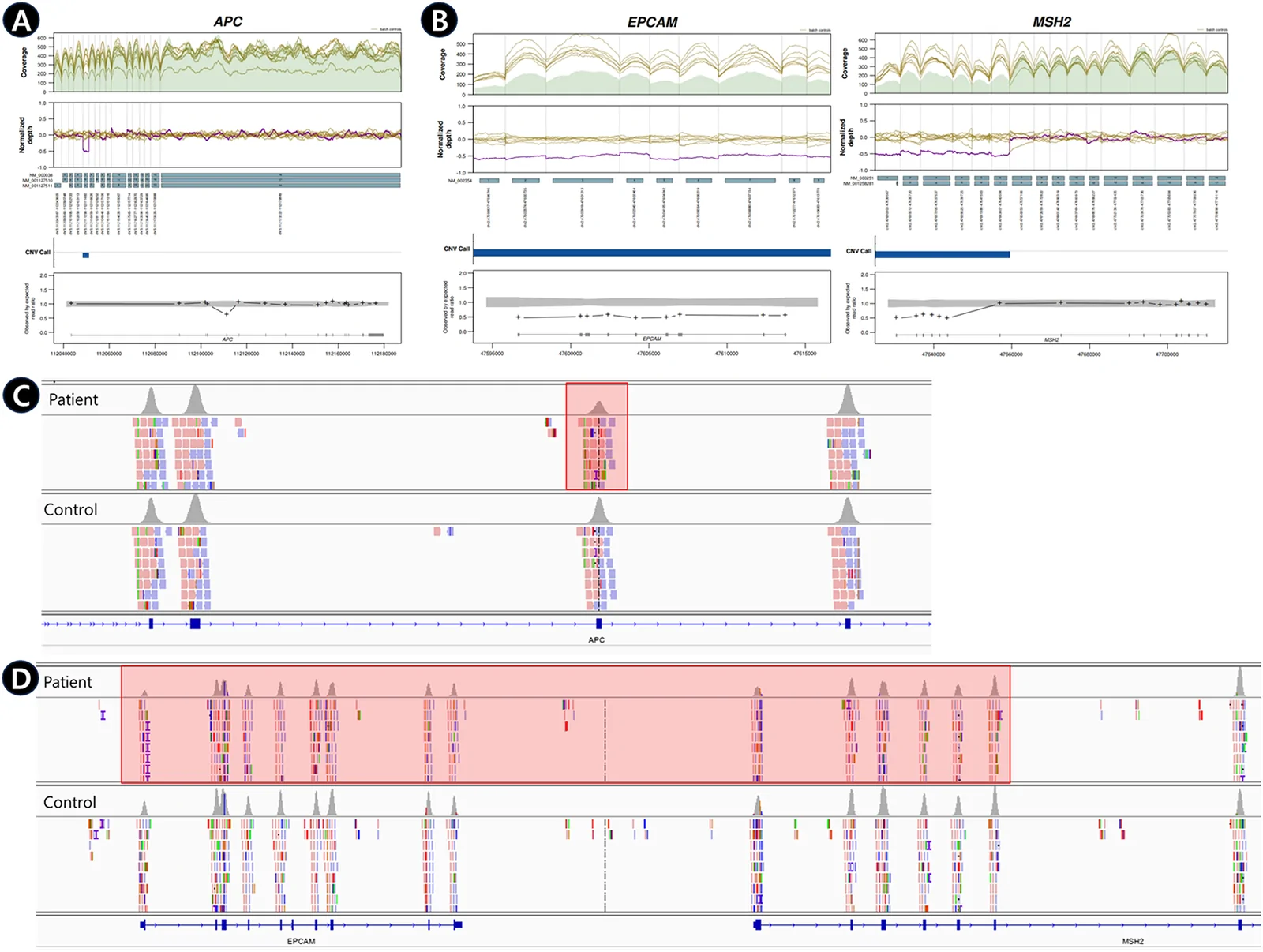
Pathogenic/likely pathogenic copy number variants (CNVs) identified in this study. (**A**) and (**C**) represent the case of “NM_000038.5 (*APC*):c.(422+1_423-1)_(531+1_532-1)del”, while (**B**) and (**D**) represent the case of “NC_000002.11:g.(?47596618)(47643594_? )del”. (**A**) and (**B**) show the results of copy number variants based on read-depth analysis from next-generation sequencing. (**C**) and (**D**) display the corresponding regions with copy number variants (red box) using the Integrative Genomics Viewer (IGV) software

Several methods, including cytogenetic karyotyping, MLPA, and aCGH, are commonly used to detect CNVs depending on the size and scope. MLPA is a well-established and widely used method for detecting CNVs in a single gene or a small number of genes known to cause cancer [8]. However, it is limited by additional tests required for each gene. NGS can be divided into hybridization capture- and amplicon-based methods; among them, the target capture method is more effective in detecting CNVs. It also involves other CNV detection methods, including paired-end mapping, split read, read depth, de novo genome assembly, and combinations of these approaches. To detect CNVs in this study, we used a hybridization capture-based NGS approach combined with a read depth method to infer deletions or duplications in specific regions based on coverage and depth information from NGS sequencing data. Our results showed that 18.1% (17/94) of the P/LP variants detected in patients suspected of having hereditary colorectal cancer syndrome were CNVs (Fig. 3). CNVs account for 10.8% of all P/LP variants in hereditary cancers and 28.6% in colorectal cancer [10]. Similar to the previous study, the present findings revealed that CNVs were clinically observed at a significantly high rate (18.1%). These findings are consistent with previous reports showing that CNVs represent an important subset of P/LP variants in hereditary cancer testing. Because Sanger sequencing has intrinsic limitations in detecting CNVs, hybridization capture-based NGS with read-depth analysis may complement sequence-level variant detection in patients suspected of having hereditary colorectal cancer syndromes.

MSI is a condition in which the number of repeated nucleotides in microsatellites changes on a genome-wide scale because of defective MMR systems caused by P/LP germline variants in MMR genes, such as *MLH1*, *MSH2*, *MSH6*, and *PMS2*, or hypermethylation of the *MLH1* promoter. In accordance with the guidelines from the National Comprehensive Cancer Network (NCCN) and the American Society for Clinical Pathology, College of American Pathologists, AMP, and American Society of Clinical Oncology, all patients with colorectal cancer for Lynch syndrome screening should be tested for MSI [14–16, 21, 24].

The IHC test for MMR proteins is a simple and useful method for detecting MMR deficiency [19–21]. MMR deficiency can result from P/LP germline variants in MMR genes or the hypermethylation of the *MLH1* promoter. MMR protein IHC staining is a well-known test for screening Lynch syndrome and the MSI-high molecular subtype. MMR deficiency or MSI-high status has been associated with a favorable prognosis and a lack of clear benefit from fluorouracil-based adjuvant therapy in colon cancer [25, 26]. Recent studies have also shown that it is highly responsive to immune checkpoint blockade immunotherapy in solid tumors [20, 25]. Accordingly, MSI testing and MMR protein IHC staining are established screening tools in patients with colorectal cancer, and our findings are consistent with their role in identifying patients who may require further germline evaluation.

The positive detection rates of P/LP variants were 60.4% and 5.1% in the MSI-high and MSI-low/stable groups, respectively. Notably, in the MSI-high group, a pathogenic variant in *BRCA1* gene was observed in one case. The positive detection rates were 66.7% for cases with MLH1/PMS2 loss, 71.1% for those with MSH2/MSH6 loss, 33.3% for concurrent loss of both MMR protein pairs and 6.4% for those with intact MMR expression. These results confirmed a high positive rate in the MSI-high and MMR deficiency groups and thus validated the effectiveness of MSI testing and MMR protein IHC staining as a screening tool. However, this study was limited because the hypermethylation of the *MLH1* promoter was not tested.

In summary, this single-institution retrospective study showed that the transition from phenotype-driven Sanger sequencing to panel-based NGS with CNV analysis was associated with an increased detection rate of P/LP variants in patients suspected of having hereditary colorectal cancer syndromes. These findings are consistent with previous reports showing the genetic and phenotypic heterogeneity of hereditary colorectal cancer syndromes and the clinical utility of multi-gene panel testing, particularly for detecting CNVs and variants in genes not predicted by the initial clinical phenotype. MSI testing and MMR protein IHC staining showed high concordance with molecular findings in patients suspected of Lynch syndrome, consistent with their established role as screening tools in current clinical practice. However, because this was a retrospective single-institution study and MLH1 promoter hypermethylation testing was not performed, further studies incorporating comprehensive clinical and molecular data are warranted.

## Supplementary Information

Below is the link to the electronic supplementary material.

Supplementary Material 1

Supplementary Material 2

## Data Availability

No datasets were generated or analysed during the current study.
